# Supplementary material for: Female genital mutilation/cutting: Emerging factors sustaining medicalization related changes in selected Kenyan communities
Source: PLoS One. 2020 Mar 2;15(3):e0228410. doi: 10.1371/journal.pone.0228410 (PMC7051066; doi:10.1371/journal.pone.0228410)
Supplement: S1 File — (PDF) [file pone.0228410.s001.pdf]

FGD WITH WOMEN

I: What is the importance/advantage of FGM?

R3: It has no important because we do it just for tradition purpose.

R1: Circumcision has both positive and negative, there is no benefit for the girls it only contributes problems when they are being circumcised e.g. when they are giving birth they are uninfibulated, having problems during menstrual period it makes them not to release the blood easily that is for Fircauni and also she will have infections. And the Sunni it is a culture that we use and it doesn't contribute so much problems like Fircauni.

R4: Circumcision doesn't contribute to any importance towards girls.

I: So to your opinion is it necessary or we can leave it?

R3: There is no much problem although it is better to do the Sunnah one like cutting small piece.

I: Is FGM documented in the religion I mean in the holy Quran?

R3: Yes the Sunnah one**.**

R4: One of its advantage is in case a girl is being attempted raped the one which is circumcised will be rescue due to the re-infibulation done during circumcision and un circumcised one will be raped immediately.

R2: To my opinion it doesn't have important and girls nowadays give birth true C.S because of the FGM so I could have said they should stop the firaun one and do the Sunnah one which is cutting of small piece of clitoris.

R1: FGM is cultural thing but to me it has no advantage.

I: Thank you who else will add some something on that?

R3: We are Muslim we should we should practice FGM.

I: Are you supporting FGM or oppose?

R4, R3 and R2: We are opposing FGM should be stopped

I. To your knowledge is FGM common in your community?

R1: Currently the community has different concerning circumcision because earlier times girls were being circumcised, but currently they are not being circumcised totally.

R2: I agree the same that all girls are not being circumcised, and there was a time when my son said his daughters cannot be circumcised and I didn’t force him to do it and she isn’t circumcised.

R3: Nowadays girls are not being circumcised.

R4: previously girls were being circumcised that is total cut of clit and then sew it but currently there is total change.

R5: I circumcised all my six daughters where by the first three were being circumcised in a cruel way that is total cut of the clit and the last three were being circumcised by cutting slight part of the clit and the circumcision was done by their aunt. And the knowledge behind circumcision is to keep modesty of the daughters.

I: What problems do they face when they are being circumcised?

R1: she will feel pain, bleeding and become weak due to what she is feeling.

R2: they will feel pain and what follows is when they she meet with man they also feel pain at that time and to some point they will get infection, the infection is being caused by the blood lost and the dowry used during the time of circumcision.

R3: daughters who are being circumcised and sewed feel more pain during the time of deliveries where she will suffer a lot.

R4: daughters get more problem when circumcised like when meeting with their husbands and during the time of childbirth deliveries.

I: Where are this daughters with all those turmoil taken to?

R1: After deliveries we use to keep them at our homes.

R2: After blood loss is when they are taken to hospitals.

I: Do you think carrying out circumcision in Kenya is it accepted or unaccepted?

R1: The government of Kenya does not accept circumcision.

R2: Currently what we are aware is that the government of Kenya does not allow circumcision to be carried out and any person who does it the government can jail or kill.

R3: The government created awareness against circumcision of now we are of it and cannot go against the law.

I: What are your opinions concerning circumcision? And do you want circumcision to be stopped or be continued?

R1: Circumcision to be stopped.

R2: Agreed with the first respondent that is circumcision be stopped.

R3: We want circumcision be stopped the reason being we have seen our daughters having problems bleeding and during deliveries.

I: What are your views?

R1: I was one of the people who do circumcision but nowadays people come home looking for me to circumcise their daughters but I preached them saying I do not circumcise and explain to them the health problems.

R2: When women came seeking for circumciser I tell them even cutting the slight part of the clit is not done nowadays and it does not help your daughter we deliver our voice while at home.

R3: Am against circumcision and I preach that it is not good idea to circumcise daughters.

I: What challenges do you face when exchanging your views with the community?

R1: Misunderstanding like some of them can even abuse you and fighting.

R2: Some of the people accept and some do not take it.

I: Are there people that support FGM in the community?

R1: The parents support FGM and they take their children far away from village in order to perform circumcision.

R2: Currently we cannot finger point someone who clearly supports FGM but when people come to us we clearly tell them that we don’t carry out circumcision go away with it from this place.

R3: Previously we use to conduct circumcision but currently we don’t witness any in the village.

I: Are there people who are against it?

R1: The parents are against it and the reason being looking back on the tradition line of the fore fathers like my grandmother was circumcised and my mother too, they go away and do it in the bush.

R2: Women are against FGM, when they visit me I do give them my views and refuse to circumcise and they get far with it doing it in far places away from the village.

I: What do they do that shows they are for or against the practice?

R1: When they actually come to me wanting me to circumcise their daughters I preach against it as something that harms the girls but parents especially women take away their daughters to areas far from the village to a certain areas where they have relatives and perform the rituals, the girl is being returned while infibulated and unable to walk.

R2: When leaders in Muslims institutions preach against it also saying girls should not be circumcised.

I: What is your view of community members that support or oppose FGM? Reasons for their views.

R1: I support the Muslim clerics that preach daughters not be circumcised.

R2: I agree with the mother (R1) and we are against FGM.

I: Have u experienced or noticed a shift or change practice of FGM in your community?

R1: There is a difference between a circumcised lady and uncircumcised one during the time of childbirth where the circumcised one will pass more turmoil than the other. They shifted from total cut of the clit to a slight cut of clit and also others do not circumcise their daughters at now.

R2: There is a change in modest, the circumcised ones are more of decent than the uncircumcised one.

R3: Our uncircumcised daughter are more off in the field of love affairs (relationship) and are healthy all times unlike the circumcised ones.

R4: There is a change in the community and girls are not circumcised at now. The Muslims clerics preach against FGM and do not support girls to be circumcised.

I: Are their people disputing the reasons justifications, for the continuity of FGM in the community? What are your personal views?

R1: Everybody has his own choice some do carry out circumcision and some don't.

R2: If I talk of my area of residence people are in suit and accept that they are all against circumcision.

R3: I second (R1) and (R2) that we are against it and totally not performing anymore circumcision.

I: What can lead to FGM abandonment in the community?

R1: Due to the health problems associated with and it not being something good and not mentioned in the laws either secular or sharia that made people to preach against FGM.

R2: The Muslim clerics preach against circumcision of ladies that made people not perform anymore and also it contributed to good health when girls are not being circumcised.

I: Do you think the community is ready to accept change and work towards good ending?

R1: They will accept, because we know hundreds of girls who are not being circumcised whereby we were called and we refused to perform it and they ended up getting married at end like that.

R2: In our community they have accepted already that girls should be circumcised because the Muslim clerics have air voice against it and the health problems associated with it.

R3: We all agree that.

I: Who are those people that come up with the decision to stop FGM?

R1: The people are many and all of them don't think the same, to my opinion if people go within the area to create awareness. Mothers are week by nature and the people to take that responsibility are the fathers because of their nature of being strong.

R2: Only the parents especially the fathers can stop it.

I: Have your daughter's undergone FGM in the community? At what age was it done? Type of FGM? Person who did it? Where was it done?

R1: We use to circumcise our daughters previously but we don't circumcise them. Previously we use to circumcise them at the age of five, six, seven or eight.

R2: Type of FGM was firaoni the total cut of the clit and sew them.

R3: Persons who use to do is the fore fathers ad they are no more.

R4: They use to do it far places like in the bushes in a nomad places and return the girls when they sew them.

I: Who use to take the initiative to perform circumcision to the daughters?

R1: Earlier times women never lead men in terms of giving views, men use to give all opinions and take responsibility of coming with the idea of circumcision.

R2: Both parents take the initiative and sleep at night knowing that tomorrow their daughter will be circumcised and even saying they will call a certain person to perform the ritual.

I: Why did you choose to have FGM performed on your daughters?

R1: That was the opinion of the parents.

R2: To me they followed the culture of the community and that is why they performed the circumcision.

R3: I second (R2) whereby it was a culture of the fore fathers.

I: What is your experience with the medicalized FGM in the community? Who performs it? Where did procedure take place? Who much did the procedure cost? How long your recovery did took? Did you developed any complications?

R1: Am against sewing of daughters it will not add anything to wounded person, I don't believe.

R2: Me I also disagree with that of sewing wounded girls in the hospitals.

R3: We are not aware of doctors who circumcise girls but for boys we know. And if the girls are circumcised by doctors the recovery will be easy and no much bleeding.

I: Do you know of other families in your community that have used medicalized FGM in the community? Family prevalence for either medicalized or tradition FGM /C practice?

R1: We never heard women taking their girls to hospitals for circumcision.

R2: They don’t agree to circumcise their daughters in the hospitals rather they will take them to far in bushes.

R3: We are not aware of something like in our village.

R4: To my opinion I don’t think I will take my daughter to the hospital and she be circumcised by a man and the community at large will not allow that.

R5: To my opinion, people with money can take their daughters to hospitals if they miss someone perform for them in the villages and end up in circumcising their daughters in hospitals.

I: In your opinion do you think that medicalization re-infibulation of fgm/c will continue? How will this impact the practice?

R1: To my opinion if they are being circumcised by women we accept our girls to be circumcised at the hospitals.

R2: If i can get the means of circumcising my daughters to be at the hospital level whether the ritual is performed by a man or a woman I don’t see any different

R3: I accept if the ritual is being performed by a woman.

R4: To my opinion there will be no problem that will occur if they are being circumcised at the hospital.

I: You have said you agree

I: Whether medicalization of fgm/c will lead to abandonment of fgm?

R1: If we go around in the village and create awareness we can make change and lead to abandonment of FGM.

R2: Circumcision in the hospital can lead to abandonment of FGM.

R3: To my opinion I can say circumcision in the hospital cannot lead to abandonment of FGM because people can come up with an idea that can lead to freshen the idea of circumcising girls in the villages in order to follow suit with the hospitals (business competition).

I: What are the advantages of re-infibulation FGM/C? Advantages for the husband, wife, community?

R1: The benefits infibulation to the husband is that he can understand if the woman is virgin or not.

R2: Infibulation can make the husband understand if the virgin.

R3: I second both (R1) and (R2).

R1: It has no benefit to the woman it only adds pain and turmoil to her life lifetime. She will feel the pain when she meets with the husband.

R2: If a woman meets with a man without legal binding between them it can lead to bleeding and can easily be confirmed she had illegal sex with someone by her parents.

R3: No other benefits.

I: Who are the decision makers for FGM/C part in the community?

R1: The decision is made by the parents.

I: Do you have any comments you would like to add?

R1: My opinion is if girls are to be circumcised in hospitals they must be circumcised by a female technician than a man.

R2: My opinion is total abandonment of circumcision as initiated now, no more circumcision.

R3: I second (R2) we should not circumcise girls anymore.

R4: As said by Muslim clerics we came from slight cut (Sunnah) to no circumcision and we support the decision we don’t have to perform FGM (Firaun)

I: Who will add something before we wind up?

R1: There is a change in FGM and it is not like before. Therefore I support Sunni way of cutting.

R2: I totally support abandonment of FGM in the community.

R3: I second (R2) too, in FGM abandonment and if the community cannot abandon they retain with the Sunnah way of cutting.

R4: Sunnah circumcision to be used.

R5: We all agree the Sunni to be used.

I: Thank you so much for your time and your oppinions we are done with discussion.

All the respondents: you are welcome.
