# Supplementary material for: Female genital mutilation/cutting: Emerging factors sustaining medicalization related changes in selected Kenyan communities
Source: PLoS One. 2020 Mar 2;15(3):e0228410. doi: 10.1371/journal.pone.0228410 (PMC7051066; doi:10.1371/journal.pone.0228410)
Supplement: S2 File — (PDF) [file pone.0228410.s002.pdf]

KII No: 075302

Gender: **Male**

Position: Government Officer

Years: 46

Marital status: Married

Number of children: 4boys, 3girls.

Lived in kuria since birth.

Language undertaken: English, Swahili, Kuria.

I: Please tell me a little bit about yourself, your role and position in the community.

R: I am an opinion leader and have been working with my community almost all my life. I have lived in this community almost all my life, he was born here brought up among the Kuria community and Kehancha is where I live.

I: To your knowledge is FGM/CUT common in your community?

R: "Mmmmh "Yes .it is almost 92%it is as if almost every household Christian and non-Christians perform it. Because if we take it literally, in Kenya Christians are 85% the rest are either Muslims or pagans so it means FGM/CUT in kuria is 92%. this only means all Christians do it and only add the very few pagans hence that makes it 92%.

I: What is the principle type of FGM/CUT experienced in the community?

R: It is a right of passage done to young girls it is used as a gauge of graduation from childhood to adult hood. They cut the clitoris.

I: Where do people go for FGM or where is it done?

R: In a secret place and in a home next to a home of a council of elder who was considered to be of a high rank in the community. He must be a family man the place best known as "ikebega".

I: What is the justification in your community?

R: Kurians believe unless the girl is cut you cannot be married locally. You are considered dirty if not cut. If you are cut the community considers you a complete woman who can handle many things including community traditional ceremonies. They are not relevant, they are outdated kind of beliefs that should be uprooted from the community, but other members

consider it still important for one to qualify in the society. You can find a woman of 80years if not cut is called a child “mtoto”.

I: Do you think the justification for the CUT by the Kuria people are relevant?

R: Yes.to the majority of the people in my community, their justifications are relevant. Groups pressing for the continuity include the council of elders who value it, most as their culture and mostly since they are the ones who celebrate most when girls are cut. Women are majority, mothers and grandmothers. It is considered a religion which must have followers.i.e. Majorly women, old grandmothers then the council of elders.

I: Are you aware of any health impacts of FGM and were the victims go when they experience complications?

R: Yes, there health impacts of FGM. We have midwives the “kwaks”, the nurses from TZ who conduct maternity wings in their houses not actually associated with facilities with qualified doctors, where the inject anyone anything to stop the bleeding. But for the traditional elders in case of excessive bleeding, the use herbs, you are caned on the head with a stick of the” omokoma” they believe omokoma can stop excessive bleeding. Also given soft fibers from tress as you chew and swallow bit by bit. Council of elders also believe whatever the amount of blood you lose you can’t die since according to their rituals if the gods accepted their sacrifice nothing will go wrong. When a cut girl died you are not buried, you are thrown in a forest in the next clan and if the other clan finds you they will carry it and throw it back to your community. You were considered a bad omen.

I: What is the legal status of FGM in Kenya and the views of the community regarding the status?

R: As a person am aware that there is FGM ACT, the children’s Act cop 87 established in 2001. The FGM Act of 2011 has criminalized FGM in Kenya. But the community knows very little about it since the government has done very little awareness neither the partner organization working around nor local organization have not a good job in educating the public. Out of ignorance and illiteracy the document has remained a document of the few, the elite community. A case of a FGM circumciser, she is arrested and is surprised that there existing laws for such a case. If you explore deeply with other members of the community, they say they were not involved during the establishment of the document. I would suggest that the chiefs should be empowered to distribute the right information to the community at the barazas. The government should enlighten the entire community and not the eight alone. The so that he can educate the public. Recommend chiefs should be empowered about the Anti- FGM act and not remain the document of the alight community but made know to the whole community i.e through chief barazas.

I: What is your personal view regarding FGM/CUT?

R: FGM should be abandoned and uprooted from the community, I am not happy seeing Kurian’s considered to be thief’s on the anti-stock theft, RDU unit, JSU are diploid.
When cows are stolen the government will bring all types of forces from the police and when more than three hundred girls have been cut we don’t see any police. When the girls’ organs have been tempered with the girl should be protected.

I: Are your views consistent with the general community?

R: No. these are personal view and the community will want to maintain their culture. I.e. it is a culture without vision, an outdated one that need to be updated.

I: Are there people in your community that are against FGM?

R: Few people, pastors of some specific churches like SDAs and some Pentecostal churches people who have gone to school and interacted with other community of this county and those that value education to FGM.

I: What do they do to show that they are against the practice?

R: Some of them openly take about it in public you can see their child who have not been cut.

Some work with are advisory council for children services.

I: What is the view of the community members about those individuals who are against FGM?

R: They see them as traitors, who love other peoples culture than theirs.

They ask which is this culture you want us to join and leave our culture, sarcastically.

I: Do you talk about FGM with your spouse, your wider family and others in the community?

R: I had served in the area advisory council, also am an Anti-FGM crusader, it has gone beyond the extended family.

I and my wife would consider ourselves as role models our girls are not-cut and we have convinced our neighbors their girls are not cut too.

I: In your own view what will lead to FGM/CUT abandonment in your community, give me the reasons, drivers and the time line.

R: To empower families to take their children to school.

The churches practicing under one accord to come up with though stands on those who cut.

Encourage young men in churches to marry only the uncut girls.

Encourage girl child education.

We should find ways of motivation girls who are not cut to be assisted in their enrolment in schools.

Build/establishment of boarding school i.e. Taranganya Girls who attract other girls like from the Lou community who don't cut they become role models to the kuria girls at cutting is not important.

I: Do you think the community is ready to accept change?

R: It is not an easy thing they are not ready, they should be forced and sensitized on negative effects of FGM.

I: Who are the key decision makers?

R: Approaches: Educating girls without discrimination to give opportunities once they finish school as men to marry uncut girls

Middle Aged women who are always in the for-front to take girls for the cut school be sensitized.

The law enforced should be given the for front to take girls for the low enforced should be given the for front and taken serious and corrupt judiciary should be put out, since it is discouraging.

I: Is the community aware of the programs on the ground that are campaigning against FGM eg: CBOs, NGOs etc?

R: View them as gate crushers who come to interfere with their norms.

The only program wildly spoken, that should be emphasized and should be put in place is the sensitization of the Anti- FGM Act.

I: Are your daughters circumcised?

R: No. my two daughters now grown up between 22years and 25years are not cut, and no one has bothered me as to why he has not cut the girls.

Those who claim social pressure to me are pretenders.

I: Apart from yourself who else supported the decision that your girls should not be CUT?

R: My wife supported me the girls not to be cut and the extended family learnt from me.

I: What is your personal experience on medicalized FGM/CUT have you had an experience with it?

R: Somehow yes, In the recent years the mid-wifes from Tanzania have some cheap theology. Since according to them traditional cutting is against in churches the community feels when

the girls are taken to the nurses it is no sin, that it is only sin if cut traditionally that is why I am calling it cheap ‘theology”.

I: Where were this practices taking place?

R: In the houses of the nurses.

I: How much does the procedure cost?

R: About Kshs.500

I: Where there any health complications?

R: They were but in most cases what they have been doing is reduced style ‘CCM style’ they call it that because it originated from Tanzania “*wanakataa kidogo’’*

I: Do you know families who prefer medicalized FGM than the traditional FGM?

R: Yes, I know both that prefer traditional and those who prefer medicalized.

They are those who believe that when they see a nurse the cut is complete, and there are those who believe unless they are cut traditionally it is not complete.

I: With your opinion do you think Medicalization of FGM will continue?

R: Yes, with the burning of the physical celebrations in kuria, it will increase the medicalization since the nurses would want to do it secretly in their houses like the kisii. You know even the percentage of kisii cutting is higher that the Kurian. But since the kurians celebrate in the public they are considered the worse. Therefore, FGM will increase from what we are experiencing today.

I: Do you think medicalization of FGM would lead to abandonment of the Traditional FGM?

R: Yes, it will lead to abandonment of the traditional FGM/CUT since we won’t be able to know the cut and those not cut.

I: Do you have any comment/opinion you would like to add on the discussion?

R: To me my recommendation, the finding done by the researchers should be shared across and locally for those who would like to establish an FGM/CUT project, let it not be a Nairobi Document but shared to help the locals. Thankyou.

I: Thank you for your time it was good having you.
