# Supplementary material for: Female genital mutilation/cutting: Emerging factors sustaining medicalization related changes in selected Kenyan communities
Source: PLoS One. 2020 Mar 2;15(3):e0228410. doi: 10.1371/journal.pone.0228410 (PMC7051066; doi:10.1371/journal.pone.0228410)
Supplement: S3 File — (PDF) [file pone.0228410.s003.pdf]

IDI FOR GAURDIAN

LOCATION: SENGERA GUCHA SUB-COUNTY

In depth interview numbeR: 03

In depth interview date: 10/11/2016

Ethnicity of the participant: Omogusii

Gender of the participant: Female

Age of the participant: 43 years

Marital status of the participant: Marreid

Number of children of the participant: 5 Children (3 boys 2 girls)

Other relevant information of the participant: Assistant Village Elder

I: What is your understanding of FGM?

R: Cutting of girls is a rite of passage that we do in this community so that she is not called names by her peers so that she is respected.

I: What is it exactly that they did when cutting girls?

R: To cut they would take the girl to the river very early in the morning and bathe her, then she would be taken to a stone where she would be cut and flour would be applied on her head then she would be secluded in a house for seven days.

I: As a mother who has children…..

R: **(**I*nterjects***)** My youngest daughter is two years

I: as a mother; what role do you play in the cutting of girls?

R: I took one of my daughters for the cut two years ago, she is now going to standard five.

I: At what age did you take her for the cut?

R: I took her when she was six years.

I: Was your husband present or had he passed away?

R: He passed away along time ago.

I: So you are the one who decided to take your children for the cut?

R: Yes, am the one who decided.

I: So we can say yyou are the one who made the decision to cut the girls.

R: Yes.

I: Is the community aware of cutting of girls?

R: Yes, they are aware; like now it is December they are cutting girls.

I: How do you know?

R: You will hear a parent saying they are looking for money to pay the cutter and to feed people for the cutting ceremony.

I: Do they still have feasts during the cutting of girls?

R: Yes they do but secretly; not like in the past; in the past they would slaughter a cow, provide alcohol and the ceremony would start the evening before the day of the cut; the feast would continue until the next day after the girl has been cut. But today you cannot find out when they do they cut. You find out after it has happened.

I: What kind of cutting is done in this community?

R: Nowadays they bring an injection which they inject on the bean (clitoris), and then they give it a few minutes waiting for it to swell; and then they cut.

I: Do they cut off all of it or they cut off a small piece?

R: They remove a small piece they don't dig too deep.

I: How do they decide on how to cut?

R: I don't know how they decide; it also depends on the doctor who is doing the cut and the skill they have; some cut a small piece and some dig deeper.

I: So, in this community; how do they do it?

R: They remove a small piece.

I: As a parent how do you tell them to do it?

R: I just tell them I have brought my child and they should do their work.

I: Where do they do it; do they do it in hospital?

R: No; you bring them to the house, they come and put the girl on the table and perform the cut.

I: Do they do the procedure in the house?

R: Yes.

I: Why do they do the procedure in the house?

R: Because they do it in a place that is secret; outside they can be spotted by someone.

I: At what time do they do the cutting?

R: Nowadays they don't have time; they come at any time; not like before when it was done before 7am.

I: What was the reason why they did it very early in the morning?

R: Because they knew that very early in the morning blood had not fully circulated in the body and the girl would not bleed a lot; nowadays when they do it at any time some girls bleed to death.

I: So as you have mentioned today it is being done by health professionals; why do they do it at any time despite the bleeding?

R: Because its like they are stealing so if they come to the homestead and there are people they will go back and come when there are no guests. I prefer how they did it in the past because it would be done very early in the morning and you would go home without bleeding too much.

I: What is the main justification of cutting girls in this community?

R: There is nothing; I don't see the value.

I: You mentioned it was for respect?

R: Yes; in the past they used to refer to a girl who is not cut as a Luo; so we used to do it so that the girl is respected.

I: Is there any pressure at the moment to cut girls so that they are respected?

R: It is a personal decision because one will decide to take their girl for the cut because you have seen others taking their daughters for the cut; so that you are respected as a parent. If you don't take your daughters for the cut you find people calling your home a home of Luos.

I: When they say that does it prevent your girls from getting married?

R: No; how will the man know if the girl is cut or not; they will find out later or she will find someone who wants a girl who is not cut; or when she is going to deliver is when they will find out.

I: So, does the fear of people finding out that a girl is cut prevent them from going to deliver at the health facility?

R: Yes; sometimes it is a problem.

I: But getting married; does it put pressure on the girl to be cut?

R: No; it is during delivery because we go to the women who assist in delivery in the community; they are the ones who will start talking “so and so’s wife is not cut”. That is why some people who are not cut prefer to deliver at the health facility because it will remain between them and the doctor.

I: Does the church present any push for the girl to be cut or not to be cut?

R: No.

I: In this community are there groups pushing for girls to be cut?

R: No; not presently; it was only in the past.

I: Which groups were pushing for girls to be cut in the past?

R: The grandmothers; when we would visit them they would make fun of us if we were not cut saying “why are you sleeping in your father’s hous and you are not cut, stay here we do the cut”.

I: Does that mean it was wrong to sleep n your father’s house if you were not cut?

R: In the past; not now.

I: So currently there are no groups pushing for the girls to be cut?

R: No; now it is a personal decision.

I: When you cut your daughter; did you have a feast?

R: When I did it the first time there was a feast but not for the others.

I: How did you do it?

R: I called my aunts and told them I had a visitor (the doctor) the next day; they came and when the doctor came we closed the door and did the cut.

I: Were there any other children or was it your daughter only?

R: Only my daughter; and I told her to hide in the house until she healed.

I: If her father was there would she have stayed in the house?

R: No she would have stayed in the kitchen or with the grandmother.

I: Did your aunts give you anything?

R: No; not like in the past when they would give you presents like sugar or flour; today they will not give you anything or maybe they can give you KSHS200.

I: How much did you give this doctor?

R: KSHS 600.

I: Did you bargain or was this a fixed price?

R: Because I wanted my daughter to heal well; we discussed and he said that for the procedure the charges were KSHS300 and for him to follow up on the child's healing the charges were KSHS 300.

I: So he kept coming to check up on the girl?

R: Yes he came for four days; she healed in four days.

I: What happened after the four days.

R: I allowed my daughter to go outside.

I: Is the doctor a well known person in the community?

R: Yes.

I: Do you know if cutting of girls has any health impacts?

R: No; I was not aware; I just found out after we were informed in the recent seminars.

I: Were it not for the seminars; have you experienced any impacts of FGM?

R: No; apart from how they say that girls who are not cut are high tempered; in my opinion there are no effects.

I: From what you have heard; where do they go when they have complications from FGM?

R: They call the doctors to their homes secretly. I have heard on the radio of people being arrested.

I: Does that mean that the doctors doing it are experts because you have not heard of complications?

R: I don't know.

I: Do you know the legal status on FGM?

R: Yes.

I: What is it?

R: They arrest the parent and the doctor who is found cutting the girl.

I: Where do you get this information form?

R: The radio.

I: What is your view of this law?

R: People should stop cutting girls; they can continue circumcising boys only.

I: Why?

R: Because they have said that when a girl is cut they have complications; although I have not heard of complications in this community but I have heard that they have problems when giving birth.

I: How?

R: I have heard that when a girl who is cut goes to deliver; they can touch the wound from the cut and this could cause bleeding which affects the brain of the baby; also if the cut is not done properly it can lead to bleeding; so as women we should stop cutting our girls.

I: Are these views consistent in the community?

R: Yes; I have heard people saying that we should stop cutting our girls.

I: Are they just speaking or have they stopped cutting girls?

R: I don't know because I haven't heard of anyone who has cut their girls. If they are doing it secretly they dot talk about it.

I: Do you discuss FGM with your family or the community?

R: Yes; when people suggest they want support with the cutting of their you will say you don't want to be arrested; but after the teachings we received recently we discuss and agree that it is best to stop.

I: Are these discussions among your friends only or the community as a whole?

R: We have women groups where we meet and have the discussions.

I: What about in your home; do you discuss FGM with your children?

R: Only the girls not the boys.

I: Is the community ready to stop FGM?

R: Yes; recently there was a family that the husband and wife fought and separated because the wife and her mother wanted to take one of the girls for the cut and the father did not.

I: How can FGM be abandoned in this community?

R: We should keep telling people there are no benefits of cutting the girl.

I: What is it that shows the community is against FGM?

R: Anyone who is found doing the cut should be arrested; but I have not seen anything that shows they have stopped. We said that since the government has said we should stop; we advice one another not to do it. We are still investigating to see if anyone is doing it.

I: So there is no evidence if people have stopped?

R: No; that is something that can be done in secret and people will not find out.

I: Currently; it is the cutting season have girls disappeared to visit relatives?

R: No; not like before when they would go to their relatives for the procedure; now all the children are around. There is no proof that they have stopped anyway.

I: so, in your opinion how can FGM be abandoned in this community?

R: They should keep educating us on the impacts of FGM so that people can stop.

I: Have you noticed any shift in how FGM is done?

R: yes; now they are doing it the medicalized way; they use an injection; before they did not use any and the cutter did wash their hands.

I: Are there any traditional cutters doing it?

R: No; only young people are doing it and they come from the hospital.

I: What profession are these people from the hospital?

R: We don't know because you are referred to the specific 'doctor' who is doing it; we don't know if they are specialized in a certain field or level.

I: Transposition in the audio to a different interview.
