# Supplementary material for: Female genital mutilation/cutting: Emerging factors sustaining medicalization related changes in selected Kenyan communities
Source: PLoS One. 2020 Mar 2;15(3):e0228410. doi: 10.1371/journal.pone.0228410 (PMC7051066; doi:10.1371/journal.pone.0228410)
Supplement: S4 File — (PDF) [file pone.0228410.s004.pdf]

<u>IDI with a 34 year old father at Kawangware</u>

I: Now, eehhhh, we will start with the number of years you have

R: I have 34 years

I: How many kids do you have?

R: 3

I: How many boys?

R: 2 boys and one girl

I: Which church do you worship?

R: Catholic

I: For how many years have you stayed in Kawangware?

R: Here in Kawangware?

I: Mmmmm

R: From two zero….(*pause*) five, six up to now, how many years are those?

I: Two zero six?

R: Seven, eight, nine, ten, eleven, twelve, thirteen, eeehhh around ten

I: Yes, 10

R:Aaiii,(*looking perplexed*) years are flying (*laughter)*ehhhh

I:*Laugher too*, two zero six by the way is 10 years

R: Years have flown like…….

I: Now, if somebody asks you who Douglas is, what will you tell him?

R: Mmmmm, Douglas otwori?

I: Who's Douglas Otwori?

R : Mmmmm (*Pause*), Douglasotwori is a person,,,,(*pause)*,,,,,of,,,,,(*pause)*

I:(*I interrupt*), like what do you do, (*pause)*,,,, where is your home, how is it that you're in Nairobi, what made you come to Nairobi, basically that

R:*(Pause*),,,ok, Douglas son of otwori, is….is… a person

I:Eeeeh

R:*Eeehhh*, I came to Nairobi

I:Mmmmm

R: I have stayed, I do businesses here and there

I: You do what??

R: I do business here and there

I: Oohhh, businesses like what and what

R:Ok, I have done many, I once did security

I: Mmmmm

R: Even *mjengo* (construction worker) I have done

I: Mmmmm

R: Currently I am a driver

I: Ooohhh, Of trucks or taxi?

R: Ahhh no no, I have my own car I drive

I: Oohhh, as a taxi or…..*pause*

R:Yeah, as a taxi…*pause*

I:So we can say that, that is per your perspective, now if I ask the community, who is Douglas Otwori, what will they tell me?

R:*Pause*….from the community?

I:Mmmmm

R: I think they will tell you how they know me,

I:As in what will they say about you, as in you see…..for example if I ask the area chief, let's assume you're a chief,,, and then I ask the community who is Douglas. They will tell me Douglas is a chief, and he does for us this and this

R: Mmmmm

I: There's a job you do to sustain the family, and then there is what you do that issignificant to the community. Now what will the community say is your significance to them?

R:As in the community in the rural area?

I:As in here here, here in Kawangware.

R:Ooohhh, here they know me as….*pause*… what I do is that to drive vehicles.

I:Oohh, driving vehicles.

R:Yeah, that's what I do

I:Nice, …as you think, here in Kawangware, is female cutting common?

R:Female cutting, as in to be frank I don't know because here we live different tribes, so you can't know if people are circumcising or not…..you know,,,, *Pause*….*shaking head*…it's not easy to know. You know this days people hide, so you can't know what somebody is doing in his home.

I:As in what of the way you think?

R: For cutting it's true they are being cut

I: So where do they take them for cutting?

R: You know you find that they know doctors, (*raising voice*)…you know it is the doctors who know how to cut… so they tell them to come to their homes, cut the girls at home and that story ends there.

I: So what of you, is your daughter cut?

R: No, she has less years, and am not intending to cut her

I: What do you think is reason for circumcising girls?

R: Ok, you know the time we were growing up, cutting was very common, *eehhh* and according to cutting they used to say that when you cut a child, the child changes from childhood to adulthood, and another thing is that,, *eehhhh*, as in they reduce something called sex, therefore the child will not be somebody who is running around, you see ?And then this cutting, tended to bring

people together, and people come to celebrate, eat,….I think that's still the reason why kids are cut.

I: Ok,

R:As in somebody moves from childhood to adulthood

I: Ok, like for now are there groups encouraging people to cut girls?

R:*Aaaahhh*no, no, right now it was illegalized

I: And what about at home in the rural?

R:At home? As at now you see the thing is illegalized, but according to the Kisii culture, there are people even if you explain to them is bad (*uncompleted statement*),,,,but in the real sense, those people who are educated and the way the government has banned it, there are those who are scared but now do it secretly. So they do it, only that they do it secretly.

I:*Ooohhh,* do you now any problems that come with cutting of girls?

R: Ok, as we've moved on and been educated, there are problems. We've read and we've known that there are problems.

I: Like which ones?

R : One of the problems I hear, is that when you are about to give birth, it normally brings problems because of the scar around the clitoris, and then…*pause*..I also hear that bleeding becomes excess, and the urge for sex reduces,, and you know marrying somebody who sleeps like a cow is a also a problem ..*Laughter*

I: *Laughter*, that's true, and then, when girls get such complications where are they taken?

R: Ok, you know, most of times if the cutters are unqualified, and it becomes worse, they take the girl to hospital, there's no other solution.

I: Do you know the law governing female cutting in Kenya?

R: Yeah, it's illegal

I: What of the community?

R: Ok, there are some people….they know but they do it because of their culture,, you know there are people who follow culture up to this time, even if you tell them anything they can't change

I: *Oohhh,* so for you personally, how do you see female cutting?

R: Ok, for cutting let it be stopped, that thing they used to say that when a person is cut that is when you change from childhood to adulthood…ok… they believe that once your cut, you're taught some things during the time of seclusion; how to live with your husband, how to take care of family but nowadays these things are taught In school, everything is taught. So still, I don't believe that cutting is good ….and saying that it finishes sexual urge,, *clicks,,,* it depends , it depends

I: Are those views of yours in tandem with other community members?

R: Ok, you know in these days they are not as common but during old days they were, but they are also inclined to the old generation

I: Do you speak about cutting to your wife, friends and the community?

R: Ok, for me I speak with my wife and some few friends of my age group, we tell each other that, that cutting was due to stupidity, so kids are not meant to be cut so, so we talk about it.

I: Are there problems that come with talking about it, as in, do you find any issues like shame arising.

R:Ok, you know it goes with age, you can’t go to those old peoples and mamas and start telling them about issues surrounding cutting, and start telling them that kids are not supposed to be cut. In that case you’ll not be in good terms with them. But if it’s for your age group, those who’ve read (educated) those who know about it…for them you can talk to them till they understand, but you can’t talk to the old regarding cutting.

I:So are there people in the community who support female cutting?

R:Yea, there are some, there are some, but those old mamas. You know they are also scared,,, not that they do it openly,, they already know that they can be arrested, but from their hearts, they are just being forced not to campaign for cutting. *Laughs*, for them it has not hit them that they are not supposed to cut.

I: So why do they support?

R: Still they believe that after somebody has been cut, there’s a change from childhood to adulthood, especially the way they were being taught when under seclusion. Now that is the reason some don’t want to stop.

I: So what do they do to support their beliefs?

R: They cut in secret.

I: Personally what do you think of these people who support cutting?

R: Ok, these people who support, because, you know this thing is in their culture. So for you to change the person, it will take time. And now these people who support it need to be educated.

You see, let them be taught, the good the bad. After consistent and repetitive teaching, but not that system where they tell people-don't cut children, if you cut, we will arrest you-now you see in that scenario they haven't helped. It's better for somebody to go to them and tell them that cutting is like this and like this-*gesturing via use of hands*-the effect it brings is this, the disadvantage is this and the advantage is this, so that people understand. Even when somebody is a thief and you want to help the person, it's not to arrest the person and take him to jail. Sit the person down and tell him stealing is bad because it can bring you such and such, so the person will understand. So for me, if the people who cut are educated instead of being threatened-as in if do this, you will be arrested. Now you see if you threaten me, I will do it secretly, you see. Now that is what they can do

I: Have you noticed any changes today in terms of cutting as compared to the old days? Like is there a difference how cutting is done?

R: No, I don't see any difference as such

I: You said people got cut as a passage and to reduce sex, are there people objecting to those issues?

R: Yeah

I: What do they say?

R: It'sbecause, if somebody is bad, she is bad. There are those already cut but still they are spoilt. They are high sex, you can still get those one's who is not cut is normal. So to me, I don't see any effect. So it does depend with somebody's personality

I:So which people cut in today's world?

R: The Kisii cut

I: As in, the people who cut the girls, who cuts them?

R: Still it's the doctors and there exist some old women who know how to cut, the doctors themselves cut the girls, as in lady doctors.

I: Are there differences between the two?

R: A difference, I don't see any difference as such, maybe we say the things they use for cutting are different. Because the old mamas use knives while these days, they use blades and Of course doctors use drugs as well. Not like during that time they used to do it the local way, they burn some herbs and apply to them so as to heal the wound.

I: What do you think is to be done apart from teaching, for people to abandon cutting?

R: 1st they must be taught, 2nd they should be shown videos to see the disadvantages of cutting and how people become after cutting, as in they be shown the disadvantage of cutting

I: If we do what you've said, female cutting will end after how long?

R: Ok, you know some people will change immediately, while others will take time, so it will take some years as it ends slow by slow.

I: Approximately how many years? 20? 30? 5?

R:*Eeehhh*, if at all people will also be arrested for doing it, I think around 10years, by then you can find that it has ended. You know the old are moving out and being replaced by the new generation, meaning the ones who believed will be reducing as the years go by.

I: Are there people in the community who campaign against female cutting?

R: Yes,

I: Like who?

R: People do tell, mostly those tribes that don’t cut do encourage the Kisii to stop circumcising.

I:Like which tribes,

R: Especially the luo, Kamba encourage the Kisii to stop cutting.

I: So what do they say is the major reason as in to why you shouldn’t cut?

R: Because of what I told you, problems in childbirth and reduced sexual urge. I think in most cases that is what they say

I: As per your perspective, is the community ready to abandon female cutting?

R: They are not ready but they are changing, that’s why I told you that the old generation is going and the once being taught are hiding to teachings, so they are changing.

I: Which people do you think should be used to bring changes?

R: You know the mamas should be targeted more because women are more involved in female cutting than men. You know the wife can say that she’s sending her daughter to greet relatives but in the real sense the girl is going to be cut. When the husband goes to ask question as in to why the girl isn’t coming back, the wife tells you it’s ok to say hi to aunties and finally you find that the kid has been cut without your knowledge. That means the women should be greatly involved in teachings in order to know what to do.

I: What is the perspective of the community regarding change?

R: *Aahhh*, as they are being taught, there seems to be an urge to change and because the government is also coming together with community leaders to educate people, now you see that a change will be effected

I: At what age do people cut?

R: In the old days they cut girls around eight years, nine years and ten years.

I: So where do they carry out the cutting procedure?

R: It's in the house, those days people used to wake you very early in the morning to go to the person who was known to cut and then they are cut thereafter which songs are sang to them in the road as they pass but now because of the illegality of this thing, now they cut at home in secret

I: You talked about women being key in the decision to cut, who else has capacity in influencing the decision to cut?

R: It's mainly every person who knows the goodness and the badness of circumcising, so everybody in the family participates, not necessarily so and so but mostly its women.

I: So you said tradition is the main reason for cutting?

R: Yes, tradition is what mainly brings about all these things.

I: You talked about doctors circumcising, how much do they ask for?

R: I truly don't know, I don't know how they charge

I: What of the recovery, how long does it take to recover after the procedure?

R: I think it's one week.

I: Do you think there are people using doctors to cut to this time?

R: Yeaaa, doctors do cut.

I: Do you think the cutting by use of doctors will end?

R: You know the doctors know it's illegal, but because of money, they do. But if at all the person taking to the doctor knows its disadvantages she won't take to the doctor in the first instance, so that's why I told you that the parents of the children are better placed to leave the practice.

I: So you said the chain people who help in decision making are the wife, grandparents, friends?

R: Yes. Also sisters.

I: Anything you wish to add

R: For what I can say is that the government should educate on the advantages and disadvantages, especially the disadvantages but not the use of force of arresting people, you know a person is a person.

I: That's so nice, thank you for your time
