# Supplementary material for: Female genital mutilation/cutting: Emerging factors sustaining medicalization related changes in selected Kenyan communities
Source: PLoS One. 2020 Mar 2;15(3):e0228410. doi: 10.1371/journal.pone.0228410 (PMC7051066; doi:10.1371/journal.pone.0228410)
Supplement: S5 File — (PDF) [file pone.0228410.s005.pdf]

<u>KII with Healthcare Provider at Gatina in Kawangware</u>

I: Maybe we can start by telling me a little bit about yourself

R: I am a Kisii by tribe

I: A Kisii by tribe.

R: A nurse by profession

I: How long was your training?

R: 3 years

I: How long have you practised?

R: About 10 years

I: Ok. In your knowledge is FGM common in your community?

R: It's not common but traditionally kisiis used to cut their girls but nowadays they are being educated so many have stopped it.

I: So many have stopped?

R: Many have stopped doing but there is still a few who practice it in secret

I: And those who are doing it in secret, why do they do it?

R: Because of those traditional beliefs, in that if a girl is cut the urge to have sex will not be high. If she has not undergone the cut the urge to have sex is high but it's not true according to current research those who have undergone the cut and those who have not are the same.

I: Ok. Alright. So what was the justification of practising FGM in the community?

R: It was their culture

I: Culture?

R: Yes, their culture

I: In your knowledge are there some groups in your community who are supporting continuation of doing FGM?

R: I don't think

I: Ok. Good, alright. Are you aware of any health impact of FGM?

R: Health impact! I know that one of during delivery those who have undergone the cut do get complications. But for those who not undergone the cut they don't have similar complications

I: Eh

R: Another problem is their health.

I: Eh

R: But mostly the one I have stated previously is the major problem.

I: Ok. Now when they get those complications during birth where do they go?

R: Usually in the past people used to deliver in their home but nowadays they deliver in hospitals.

I: Eh

R: Most of the people go to hospitals.

I: Eh

R: It's only a few the primitive ones who delivers at home or those where the health facility is far. But most go to the hospital.

I: Ok. Alright. What is the legal status of FGM in Kenya?

R: Legal status?

I: Yes, Legal status

R: Eh

I: Am asking how legal issues are concerning FGM in Kenya?

R: According to Kenya law nowadays

I: Eh

R: It has burned practising FGM

I: Ok

R: The government is enforcing law that the one who is found practising FGM is prosecuted according to the law.

I: Eh. What your view on the legal status?

R: My view is, there is no need to cut the girls. The one who is found practising to face the rule of law. It the right of the girl not to be cut.

I: Ok. It's their right!

R: Yes

I: Alright. As a person what is your view on FGM?

R: My view?

I: Yes, your view

R: My view, all matters to do with FGM to get rid off completely

I: Eh

R: There is no need because even in the beginning when God instructed Abraham to get circumcised he didn't instruct Sarah to be cut.

I: Eh

R: hence it's only men who should be circumcised not girls.

I: Eh

R: So the practice should be done away with

I: Is your view consistent with the community view?

R: According to the community, like for example those who understand are those of recent ages but the old folks to explain to them is very difficult but nowadays everybody is being enlightened about FGM.

I: Eh. Like elders of who old?

R: People are moving from analogue to digital, they have started being enlightened

I: Eh, You said elder of how old?

R: The elderly are the one who are difficult to understand because of their tradition

I: Eh

R: The current generation has no problem to understand

I: Ok.

R: If you explain to them they understand

I: Ok

R: Did I answer the way you expected me to?

I: yes, you answered well. Generally do people talk about FGM? Either with their spouses, their families, friends as a community. Yeah do they talk about cutting the girls?

R: As I have explained to since we started

I: Eh

R: Most people don't concern themselves with FGM issues

I: Eh

R: Those issues of going to cut the girls

I: Eh

R: Those who are doing it are doing it in secret but not like in the past where people used to plan together for girls to be cut.

I: Eh

R: Most of the people have stopped practising. Even if you go to where I come from

I: Eh

R: The culture of Kisii used to cut their girls but nowadays it's not common

I: It's not common?

R: Eh, they have been educated they’ve understood

I: Are there groups who oppose FGM?

R: yes

I: Which ones?

R: For example, there are women when the go to hospitals like taking the children to clinics they are taught

I: Eh

R: Mostly I have not seen groups moving around but the education takes place mainly in hospitals

I: Eh, Training against FGM?

R: Eh

I: Are there some organisation you have seen campaigning against FGM?

R: I don’t know that well because am not usually there (in his community)

I: Eh

R: But here I have not seen anybody visit me like the way you have done.

I: Eh

R: But I do hear there do exist such organisations against FGM

I: Ok. In your view, have you experienced a shift or a change in the practice of FGM in the community?

R: Change? Eh, the change that I have seen is the one I have told you that in the past people used to practice FGM but nowadays people have stopped the practice

I: Eh

R: People have moved on and are thinking about other issues

I: Eh

R: Things to do with FGM is forgotten, that the change I have seen

I: Are there some people who have stuck to the practice

R: As I have told you there are there but few

I: Eh

R: Those who do it in secret not in the open

I: Ok. Not in the open?

R: Yes

I: So there is that shift of doing it in secret?

R: Yes, and those who do it practice in fear because if it known they can be prosecuted

I: Eh, So those practising can't be known?

R: It's not easy to get them because they risk doing it

I: Eh

R: If they are caught they have to answer and nowadays the sub-chiefs and chiefs are there to follow up such cases

I: Eh

R: If there is rumours you have done it they will catch up with you

I: Ok. In your view are there some health providers who help those practising in secret?

R: Yes

I: Eh

R: You know! They talk with those they know

I: Eh

R: Not with those they don't know. Instead of cutting the girls they should circumcise the boys

I: Eh

R: You know, one can bring even 20 girls and they don't get injured

I: In my community they used to target the clitoris and cut a small part of it.

I: Eh

R: But there some places like Kipsigs they destroy the genitals completely but in my community it's different.

I: Ok

R: you can't know if a girl has been cut

I: Eh. But there this aspect of health care providers doing it

R: I have told you that not something a person will do in the open they will do it in secret

I: Eh, it's difficulty to know them?

R: Yes. If someone want it they go to the health provider they know mostly the female, not men

I: Female

R: Yes, the female

I: Are they nursing officer! Which cadre? Who do they target?

R: In the past they used to be cut traditionally by the elderly women

I: Eh

R: Nowadays if you go any nurse or health provider how understand the act he/she will do it for you

I: Eh

R: But it’s the female health providers not men

I: It must be a woman? A lady

R: Yes. Not a man

I: And you think that is being practised In Nairobi?

R: I don’t think

I: Eh

R: I have not heard of it here

I: ok. But back home

R: Yes in my community it’s there

I: Alright

R: in the past there were many who wanted to do it even here but recently I don’t know

I: Eh

R: Those who want to do it plan like in December and go to their communities to do it, you can’t know it.

I: Eh

R: That an example am giving

I: Ok. But as I was walking I talked to some parent who told me that if you need you just go and it’s done to you.

R: Eh

I: What’s your take on that?

R: As I told you most are travelling home like now during Christmas. During this period the act will be done after Christmas they come back already cut.

I: Eh. Have noticed some change where some families says their girls will not be touched (cut)

R: There are so many families. Like in my community there are many families me being one of them

I: Ok. In your view, what can help us to eradicate FGM?

R: human beings are difficult, even there were defiant to the Lord because when sin came in everything went wrong.

I: Eh

R: the only thing to do is to continue educating the public

I: you suggest we educate?

R: Yes

I: Why do you think education will help?

R: It will help those who do not understand easily. When sin entered human brain was corrupted

I: Eh

R: hence to understand is difficult theta why they need to be educated continuously

I: Eh

R: But it will come to an end

I: How long will that take?

R: It depend, I hear them in the media. People like Maasai saying they can't abandon their culture.

I: Eh

R: in my community I can clearly say they have improved

I: Has education contributed?

R: Yes. It's education which has helped to stop FGM

I: Ok.

R: Am talking about my community but the rest are difficult, even you can see they are difficult but at long last if the government enforces the law we shall have an end to it

I: ok. Are people in your community that are speaking against FGM?

R: Yes, there are there

I: Who are they?

R: Back at home I don't know since I don't live there but my wife says she sees them on the road as well as in churches.

I: so they go event to churches?

R: yes. Even the chiefs mobilises people

I: Chiefs?

R: Yes

I: how has the community being responding towards this?

R: They are responding positively because FGM is being eradicated smoothly

I: Ok. According to you hoe is the level of success?

R: In my area it's almost 100% although there those who are doing it in secret

I: Eh. Do you think this community is ready to change and work towards ending FGM?

R: Already it has changed

I: Eh. So it's ready? Why?

R: I have told it has changed because it doesn't happen like in the past whereby there were seasons to conduct FGM in the community.

I: Eh

R: In many families in the past the used to prepare both girls and boys to face the cut but nowadays it's only boys who are prepared to get circumcised.

I: Ok. Who are the key decision makers / key figures? That can drive this process of acceptance

R: Mostly the government has enforced law through chiefs and some health care providers who are Christians do educate people in churches.

I: Eh

R: That's what is happening

I: Ok. How does the community see this programmes?

R: They are good because they are helping them to understand the merits and demerits of FGM

I: Eh

R: They have understood

I: You may have answered this question but for clarification, you have daughters?

R: Yes

I: have they undergone the cut?

R: No, Am one of those opposing FGM

I: Ok. Going on well, we are almost there

Which type of FGM do they carry out?

R: Eh!

I: For those who practice how do they do it? I think you had told me earlier

R: I told you I see it in media like among the Maasai I see the fighting saying they won't **abandon the cut for example.**

I: My question was the type of FGM being carried out in the community

R: Eh

I: Do they cut, and how do they cut it?

R: they only cut a small part of the clitoris

I: A portion?

R: Eh! A portion. It's only the clitoris they cut not any other part

I: ok. Is to everybody who is a Kisii?

R: Yes. For those who used to in the past

I: Eh

R: Currently they have stopped it. They only target that not any other part of the body

I: Now, how does the fact that FGM is illegal in Kenya affected the practice?

R: There those who don't want to stop it, those are the ones who are pulling us behind

I: Eh

R: I don't know whether I have answered your well. Those who have accepted are moving on.

I: What is your experience on training of FGM?

R: Eh

I: Training about FGM

R: According to my experience and knowledge cutting the girls is useless (has no value)

I: Eh

R: Have I answered you well?

I: Maybe to a further question to understand where am coming from. Is there any professional training … Eh you are a nurse?

R: Eh

I: Are there any professional training that you have undergone that touches on the issues of FGM?

R: No. I have not be called to participate in such a training

I: Eh

R: Most of the trainings have been introduced in the recent years and some of the health providers are selected to do it.

I: Eh

R: As for me I have not done it

I: ok. Are people you often discuss with on issues concerning FGM?

R: According to me I discuss that with my family especially my elder

I: Eh

R: Because we sat and said if we have girls not to make them undergo the cut

I: Eh

R: But for others I won't talk on their behalf

I: Alright, thank for your time and that the end of our interview.

R: Eh

I: Thank you very much.
